# Chemical and Sensory Evaluation of Blackberry (*Rubus* sp.) Juice Concentrated by Reverse Osmosis and Osmotic Evaporation

**DOI:** 10.3390/membranes15010010

**Published:** 2025-01-06

**Authors:** Juliana Vilar, Flavia Monteiro, Luiz Corrêa-Filho, Flávia Gomes, Renata Tonon, Daniela Freitas-Sá, Suely Freitas, Lourdes Cabral

**Affiliations:** 1 Postgraduate Program in Food Science (PPGCAL), Federal University of Rio de Janeiro, Cidade Universitária—Ilha do Fundão, Rio de Janeiro 21949-900, RJ, Brazil; julianavilar@nutricao.ufrj.br (J.V.); flaviasilvamonteiro@gmail.com (F.M.); freitasp@eq.ufrj.br (S.F.); 2 Postgraduate Program in Food Science and Technology (PPGCTA), Federal Rural University of Rio de Janeiro, BR-465, km 7, Seropédica 23070-200, RJ, Brazil; lucaalbernaz@gmail.com; 3 Embrapa Food Technology—Av. das Américas, 29501, Rio de Janeiro 23020-470, RJ, Brazil; flavia.gomes@embrapa.br (F.G.); renata.tonon@embrapa.br (R.T.); daniela.freitas@embrapa.br (D.F.-S.)

**Keywords:** membrane separation processes, anthocyanin, antioxidant capacity, sensory

## Abstract

Blackberry can be considered a source of phenolic compounds with antioxidant properties, especially anthocyanins, which are responsible for the attractive color of the juice. However, blackberry juice quality can be reduced under severe heat treatments, resulting in darkened color and altered taste. Membrane separation processes are an alternative for the clarification and concentration of fruit juices, with advantages as the maintenance of the nutritional, sensory, and functional characteristics of the product. The aim of this work was to evaluate the effect of membrane concentration on the physicochemical and sensory characteristics of blackberry juice. The juice was first clarified by an enzymatic treatment associated with microfiltration and then concentrated by reverse osmosis and osmotic evaporation. Samples were analyzed for pH, titratable acidity, soluble and total solids, phenolic content, antioxidant activity, and total anthocyanins. The concentrated juices were then reconstituted for sensory evaluation. It was verified that reverse osmosis and osmotic evaporation resulted in juices with total solid concentrations of 29 and 53 g∙100 g^−1^, respectively, with slight differences in pH and acidity. Some phenolic compounds were lost during processing. The concentration of anthocyanins and the antioxidant capacity of the osmotic evaporation-concentrated juice increased 6.2 and 7.7 times, respectively, compared to the initial juice. Regarding sensory analysis, the juices concentrated by RO and EO presented acceptance percentages (scores between 6 and 9) of 58% and 55%, respectively. Consumers described them as “good appearance”, “refreshing”, “tasty”, “sweet”, or “with ideal sweetness”, in agreement with the high acceptance scores (6.2 and 6.9, respectively).

## 1. Introduction

Today, consumers demand food and beverages that are healthy, convenient, and sustainable. The environmental impact of production plays an important role in their choices. Membrane technology offers numerous ways to meet the evolving demands of today’s consumers. By improving product purity, nutritional content, sustainability, and safety, membrane processes align with consumers’ desires for healthier, cleaner, and more sustainable food.

The fragile structure and high post-harvest respiration rate of fresh blackberry are responsible for its quality loss during storage, resulting in limited shelf life and a reduction in quality and health benefits related to bioactive compounds. Therefore, blackberries are mostly commercialized as processed products, such as frozen or dried fruits, pulp, jellies, jams, yogurts, ice cream, syrups, soft drinks, and clarified and concentrated juices [1,2].

Besides its pleasant taste, flavor, and attractive color, blackberry juice is a natural fit for the nutritional juice market, offering numerous health benefits due to its high antioxidant content, vitamin C, fiber, and anti-inflammatory properties. This fruit is a rich source of phenolic compounds with antioxidant properties, particularly anthocyanins and flavonoids, that can bring benefits to human health [3,4,5,6,7,8]. Anthocyanins are also mainly responsible for the attractive color of blackberry juice. Several factors influence anthocyanin stability, including pH, light, oxygen, enzymes, ascorbic acid, sugars, sulfur dioxide or sulfite salts, metal ions, and copigments [9].

Membrane separation processes, such as microfiltration (MF), nanofiltration (NF), reverse osmosis (RO), and osmotic evaporation (OE), have been evaluated as green technologies with the potential to concentrate or clarify fruit juices without compromising their nutritional value, flavor, or quality. This aligns perfectly with the growing consumer demand for healthy, functional beverages that offer natural, nutrient-dense ingredients and clean labels [10].

Traditionally, the concentration of fruit juices has been achieved by thermal treatment. However, industrial thermal treatments may have negative impacts on nutritious components, such as anthocyanins and other bioactive compounds. This practice can also result in darkened and cooked products with altered taste and flavor [10,11]. Membrane technologies are favored in juice concentration for their ability to produce high-quality, nutritious products while offering energy-efficient alternatives to traditional heat-based concentration methods [12,13,14,15]. Recently, watermelon juice was concentrated up to 65 °Brix by forward osmosis. The membrane process has outperformed the thermal concentrate in sensory hedonic rating [16,17].

Reverse osmosis is a pressure-driven membrane separation process in which a hydraulic pressure greater than the solution osmotic pressure is applied, so that water permeates from the high (juice) to the low solute concentration. This process requires the use of high operating pressures to overcome the juice osmotic pressure [18,19,20]. Membrane processes generally consume less energy than distillation, which requires significant heat. However, distillation can achieve higher separation efficiencies for certain mixtures, especially when the boiling point differences are large. On the other hand, the main disadvantage is related to the lower concentration level that can be obtained by reverse osmosis concentration compared to that produced by thermal evaporation, since the high osmotic pressure of the fruit juice limits the efficiency of the process. In classical juice industries, the concentration levels of fruit juices range from 42 to 65 °Brix, so that reverse osmosis should be viewed as a first-stage process coupled with other processes such as osmotic evaporation.

Osmotic evaporation and membrane distillation are rather new membrane concentration processes. In these processes, water is removed by evaporation at atmospheric pressure and temperatures near the ambient temperature through a porous hydrophobic membrane. The driving force of these processes is the water vapor pressure difference, obtained by the water activity difference between the juice and an extraction hypertonic solution, in the case of osmotic evaporation. Juices concentrated by osmotic evaporation can reach high concentration levels (up to 60 °Brix), while maintaining good nutritional and sensory quality, as the driving force is not a hydraulic pressure difference [21,22,23].

The coupling of reverse osmosis and osmotic evaporation is a potential alternative for the concentration of liquid extracts and fruit juices, since it results in concentrated products with soluble solid content, like those obtained by vacuum evaporation, with less pronounced effects on the product’s quality [24]. These processes are carried out under moderate temperature and pressure, which means that besides offering technological advantages, they are also economically viable, since their energy consumption is low [25,26,27]. Zambra et al. (2015) demonstrated that the concentration of cranberry juice by membrane technologies does not affect the phenolic compounds, specifically the anthocyanin content of the juice [28].

All these results encourage the concentration of other berry juices by means of these techniques. This work aimed to evaluate the potential of coupling reverse osmosis and osmotic evaporation processes to concentrate blackberry juice. The processes were evaluated regarding the concentrate juice quality (physicochemical and sensory properties) as well as permeate flux and volumetric concentration factor (productivity).

## 2. Materials and Methods

### 2.1. Blackberry Juice Preparation

Blackberry cv. Tupi was purchased from the local market of Rio de Janeiro, Brazil. The fruits were disintegrated in a depulping machine (Bonina 0.25 df, Itametal, Itabuna, Brazil) with a 0.8 mm diameter sieve. The pulp was centrifuged in a basket centrifuge (Equipment Company, Needham, MA, USA) at 4000 rpm (2.53× *g*) and stored at −18 °C until use.

### 2.2. Processing Design

Initially, the microfiltration, reverse osmosis, and osmotic evaporation processes were evaluated separately in order to define the best operational conditions for each one.

**Figure 1:**
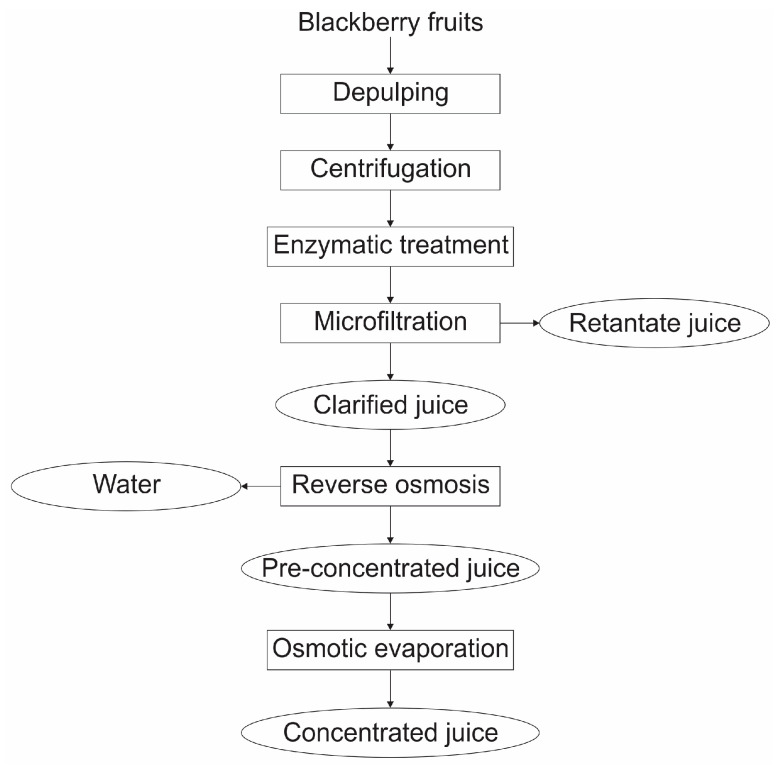
Diagram of blackberry juice concentration by membrane processes: from raw material to concentrated juice.

In the second stage, a single experiment was planned, where the same processes were executed consecutively, as illustrated in Figure 1.

### 2.3. Juice Clarification

Before concentration, the juice was clarified by enzymatic hydrolysis associated with microfiltration. The enzymatic treatment was carried out with 0.4 g∙kg^−1^ of the commercial preparation Rapidase TF (DSM Food Specialties, São Paulo, Brazil), with initial activity of 950.53 UI∙mL^−1^, in a jacketed stainless-steel tank with constant mechanical stirring (150 rpm), for 30 min at 35 °C (the better temperature for this enzyme action). All subsequent processes were carried out at this temperature.

The juice was then clarified by microfiltration using flat-sheet polysulfone membranes with 0.15 μm pore size (DSS, Silkeborg, Denmark) in a 0.36 m^2^ plate and frame module (GEA Filtration, Hudson, NY, USA). The transmembrane pressure was kept at 0.5 MPa, and the temperature was set at 35 °C to improve permeate flux value and decrease the processing time, avoiding fruit juice oxidation. In total, 25 L of treated juice was microfiltered in a batch mode. During the process, the permeate stream (clarified juice) was continuously collected, while the retentate juice was recirculated back to the feed tank.

### 2.4. Concentration

The clarified juice was concentrated by reverse osmosis before osmotic evaporation. The process was carried out at 35 °C and 6 MPa in a 0.33 m^2^ plate and frame reverse osmosis Lab Unit 20 system (DSS, Silkeborg, Denmark), composed of RO99 thin-film composite membranes (DSS, Silkeborg, Denmark), with nominal rejection to NaCl of 98%. The pump flow rate was kept at 650 L/h. In total, 15 L of clarified juice fed the feed tank, and every 15 min of the process, 1 L of juice was added until the end of the process.

The juice previously concentrated by reverse osmosis was used as feed in the osmotic evaporation process. Osmotic evaporation was carried out in a lab-scale system composed of two independent closed compartments, one for the juice and the other for the brine. A flat-sheet polytetrafluoroethylene membrane (Pall-Gelman TF200, Paris, France) with a total permeation area of 0.031 m^2^ separated the two circuits. According to the manufacturer, its average characteristics are 60% porosity, 0.2 mm average pore diameter, and 165 mm thickness. The juice was concentrated in a circulation loop continuously fed by raw juice, and two positive pumps were used for the circulation of the solutions on each side of the membrane. In total, 2 L of blackberry juice concentrated by reverse osmosis was used. The brine solution consisted of 6.5 M CaCl_2_. Briefly, 15 L of brine solution was used in order to minimize the dilution rate due to water transfer from the juice to the brine stream. The concentration of calcium chloride solution ranged from 5.5 to 6.0 mol L^−1^, corresponding to water activity values from 0.435 to 0.329 at 25 °C. The temperature during the process was kept at 35 °C for the juice and 15 °C for the brine, with a transmembrane pressure of 20 kPa, a strategy to improve permeate water flux and improve the concentration factor.

### 2.5. Process Evaluation

The membrane processes were evaluated with regard to permeate flux and volumetric concentration factor (VCF), calculated according to Equations (1) and (2):

(1) $$J=\frac{V}{A\times t}$$

(2) $$VCF=\frac{V_{R}}{V_{F}}$$

where V is the volume permeated in a time t, A is the membrane surface area, V_R_ is the final retentate volume, and V_F_ is the initial feed volume.

### 2.6. Membrane Cleaning

To clean the membrane modules, abundant water was circulated in order to completely remove the blackberry juice. After that, a sodium hydroxide solution was circulated in the system. Finally, abundant water was circulated until the basic solution was completely removed. At each new test, measurements of hydraulic and blackberry juice permeability were taken at different temperatures and pressures to ensure that the membranes were clean and intact.

### 2.7. Physicochemical Analysis

All the samples were analyzed for pH, total and soluble solids, and total acidity [29]. Total anthocyanin content was determined as described by Giusti and Wrolstad (2001) and expressed as mg anthocyanin (cyanidin-3-glucoside) per 100 g of juice [30]. Antioxidant activity was measured using the Trolox equivalent antioxidant capacity (TEAC) assay, also known as ABTS cationic radical scavenging activity, expressed as μmol Trolox per g of juice [31].

### 2.8. Sensory Evaluation

Four samples (fresh pulp, clarified by microfiltration, clarified, and concentrated by reverse osmosis and clarified and concentrated by reverse osmosis + osmotic evaporation) were reconstituted to 13 °Brix (the approximate soluble solid content of some commercial blackberry juices found in the market) by adding appropriate amounts of water and sucrose. The reconstituted samples were then presented to 96 consumers, who were asked to score their overall liking and appearance using a 9-point hedonic scale varying from 1 (disliked extremely) to 9 (liked extremely). The consumers were asked to provide up to four words to describe each sample using the open-ended question methodology. The samples were served in 50 mL plastic cups at refrigeration temperature, following a balanced design to reduce the first-order and carry-over effects.

All panelists were informed that the juice was processed, microbiologically evaluated, and suitable for consumption. Before the test, all panelists signed that they agreed to undergo the sensory evaluation.

All the juice samples were analyzed for their microbiological quality prior to the sensory analysis test and considered fit for consumption in accordance with Brazilian legislation for fruit juice. The counts of mold, yeast, and aerobic and psychotropic bacteria, the coliform group, and *Salmonella* spp., considered the main spoilage microorganisms that can develop in fruit juices, were determined according to Downes and Ito (2001) [32].

### 2.9. Statistical Analysis

All the results were obtained in triplicate and statistically analyzed by analysis of variance using the software Statistic version 8 (Statsoft, Tulsa, OK, USA, 2004). Mean difference analysis was performed using Tukey’s or Fisher’s test at *p* ≤ 0.05.

## 3. Results

### 3.1. Juice Clarification

The main physicochemical characteristics of the fresh pulp, feed (enzymatically treated pulp), retentate, and permeate (clarified juice) of the microfiltration process are summarized in Table 1. A slight increase in the total and soluble solid content was observed in the retentate fraction compared to the fresh pulp or feed (enzymatically treated). According to Cissé et al. (2005), the microfiltration membrane does not reject these solutes, and the clarification does not affect the sugar/acid balance of the permeate and retentate fractions; therefore, the refractive index may be affected by the presence of high pulp content in the retentate [33]. The retentate and the clarified fractions showed acidity and pH values similar to those of the feed juice, indicating that the membrane did not markedly affect these properties.

An increase in the anthocyanin concentration and antioxidant capacity was noticed in the retentate juice. Anthocyanins are water-soluble pigments that are relatively small, ranging from 400 to 1000 Da. Microfiltration membranes are designed to retain particles and molecules that are larger than the pore size of the membrane, ranging from 0.1 to 1 µm. Thus, anthocyanins might pass through the membrane along with other dissolved substances. But they can form complexes with fibers, sugars, or proteins, which could explain the anthocyanin retention smaller than 100% during the clarification step. Membrane structure and configuration, fouling, concentration polarization phenomena, as well as juice composition can also be associated with microfiltration retention [34].

Although the clarified juice presented a decrease in the anthocyanin content and antioxidant activity in comparison to the hydrolyzed juice (feed), this fraction can still be considered an interesting product for subsequent concentration processes.

**Figure 2:**
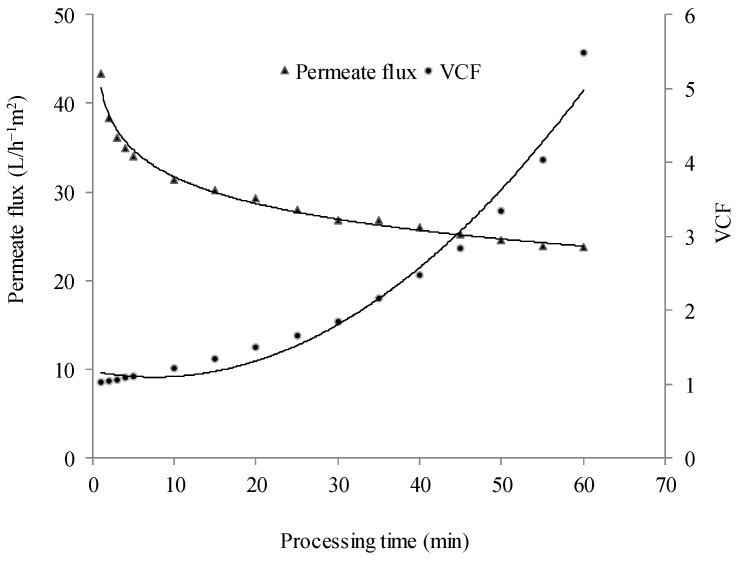
Evolution of the permeate flux and volumetric concentration factor of the blackberry juice during the microfiltration process.

Regarding the permeate flux during clarification, it is possible to observe classical decay along the processing time. The permeate flux was around 43 L∙h^−1^∙m^−2^ at the beginning of the process, decreasing to 23 L∙h^−1^∙m^−2^ at the end. The volumetric concentration factor (VCF) increased exponentially over the clarification process (Figure 2).

Although enzymatic treatment was done to reduce the juice viscosity and facilitate its permeation through the membrane, the decrease in the permeate flux may be attributed to the concentration polarization and the formation of a gel layer, which characterizes the fouling phenomenon [35]. In the case of fruit juices, the foulants are generally composed of cell wall and polysaccharides such as pectin, cellulose, lignin, and hemi-celluloses [36,37].

### 3.2. Juice Concentration

The clarified juice was concentrated by coupling the reverse osmosis and osmotic evaporation processes. The characterization of the concentrated juices obtained in each process is shown in Table 2.

Reverse osmosis resulted in a juice concentration of almost 4 times, promoting an increase in the soluble solid content from 6.5 °Brix to 24.6 °Brix. The samples showed a slight variation in the pH and an increase in acidity, anthocyanin content, and antioxidant activity due to juice concentration. The retentate fraction presented anthocyanin content and antioxidant capacity 4.2 and 4.0 times greater than those of the feed (clarified juice), respectively.

The retention of bioactive compounds depended on the applied operating conditions, but it also depended on the chemical properties of compounds, membrane characteristics, and the interactions between retentate components and the membrane surface. A slight loss of phenolic compounds was observed after the nanofiltration process was used for the concentration of phenolic compounds and the antioxidant activity of Cabernet Sauvignon red wine [38,39].

**Figure 3:**
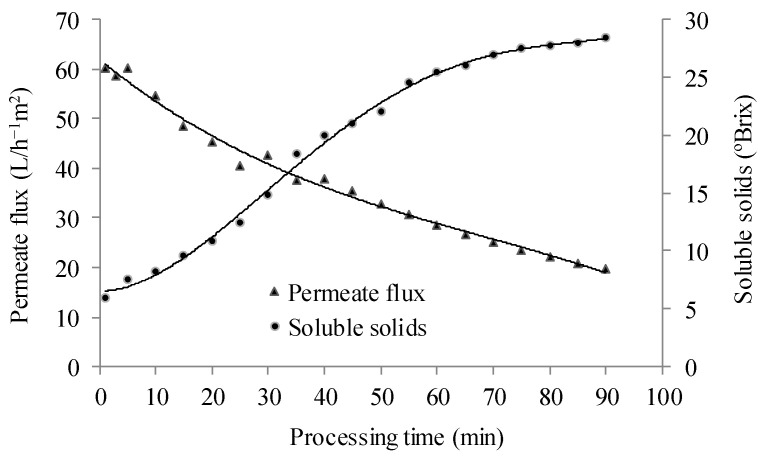
Permeate flux and soluble solid content during reverse osmosis of clarified blackberry juice.

The permeate flux during reverse osmosis decreased from 60 L∙h^−1^∙m^−2^ to 20 L∙h^−1^∙m^−2^, reaching a volumetric concentration factor of 4.9 over a 1.5 h process (Figure 3). This is a typical behavior in membrane separation processes, attributed to concentration polarization on the membrane surface at the beginning of the process (not measured), as well as fouling phenomena and the increase in osmotic pressure and juice viscosity due to solid concentration. These factors result in higher resistance to mass transfer and, consequently, a decrease in permeate flux [40].

Permeate flux in membrane separation processes is a critical parameter for membrane performance. It is well known that permeate flux is affected by factors such as feed characteristics, membrane materials and properties, and operating conditions. The reduction in membrane flux below that of the corresponding pure solvent flow over time promoted by membrane fouling leads to losses in productivity and higher operating costs as a result of higher energy cost and maintenance [41].

In the osmotic evaporation process, the juice previously concentrated by reverse osmosis was concentrated 2.2 times, reaching a soluble solid content of 55.5 °Brix. The pH did not change significantly, although the titratable acidity was concentrated around 2 times.

Total anthocyanin content and antioxidant capacity were higher than the feed juice, showing concentration factors of 1.5 and 1.9 in comparison to the juice pre-concentrated by reverse osmosis, respectively. Both anthocyanin content and antioxidant capacity had lower concentration factors than the volumetric concentration factor, indicating that part of this pigment was lost during processing, which can be attributed to oxidative reactions during osmotic evaporation, since the process was carried out for a long time (22 h) due to the small membrane surface.

The profile of the flux behavior was similar to those reported in studies about fruit juice concentration using membrane technologies with osmotic driving force [42,43,44].

**Figure 4:**
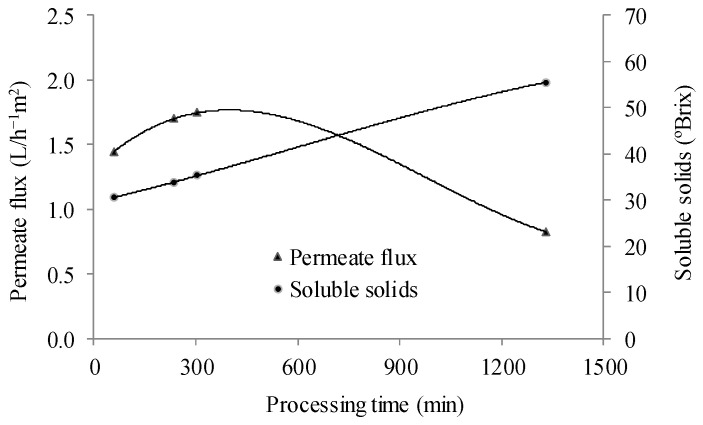
Permeate flux behavior and soluble solid content during the osmotic evaporation process.

Zambra et al. (2015) evaluated the concentration of cranberry juice by osmotic distillation, a similar membrane concentration process carried out under isothermal conditions [28]. They observed that this concentration process did not significantly affect the phenolic compounds and anthocyanin content. A considerable decrease in the permeate flux was observed after 5 h of osmotic evaporation (Figure 4). This decrease is probably due to the dilution of the brine, which reduces the driving force for the process. Moreover, above a certain soluble solid content (around 33 °Brix), the main cause of the permeate flux reduction during osmotic evaporation is the increase in the soluble solid content and, hence, in the juice viscosity.

The development of membrane processes thus requires the development of new high-performance materials, robust and reliable module technologies, and optimized process engineering and design tools, as pointed out by Favre (2022) [45].

### 3.3. Sensory Evaluation

As previously mentioned, the concentrated juices were reconstituted to 13 °Brix for sensory evaluation. The results of the acceptability test of the fresh pulp and the blackberry juice clarified and concentrated by reverse osmosis and osmotic evaporation are shown in Table 3. The mean overall liking scores ranged from 5.6 to 6.9, with highly significant (*p* < 0.05) differences.

**Figure 5:**
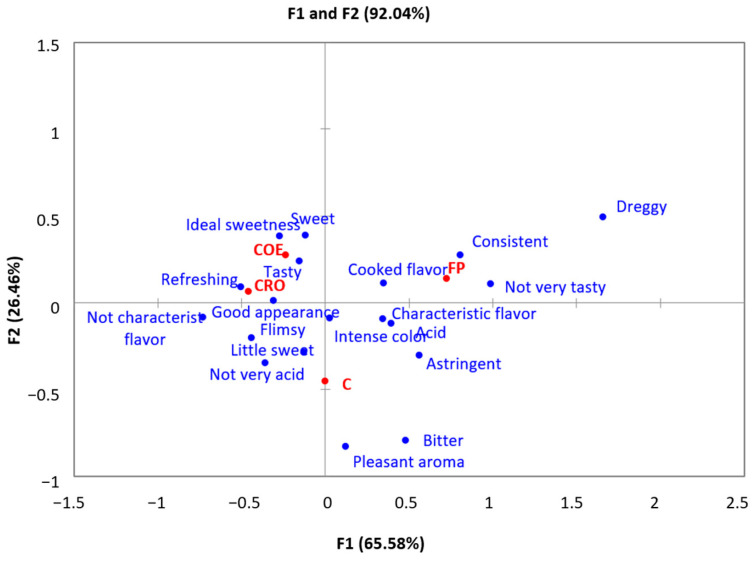
Correspondence analysis: samples (red) and consumers’ description (blue) (FP—fresh pulp, C—clarified by microfiltration, CRO—clarified and concentrated by RO, and COE—clarified and concentrate by OE).

The results of correspondence analysis (Figure 5) showed that the juices concentrated by reverse osmosis and osmotic evaporation were described as having good appearance, refreshing, tasty, and with ideal sweetness or sweet, which agrees with their highest mean overall liking scores (6.2 and 6.9, respectively).

On the other hand, the clarified juice presented the lowest score (5.6) for overall liking and was described as astringent, bitter, and acidic, despite being characterized by intense color, pleasant aroma, and characteristic flavor. This score may be because Brazilian consumers, in general, are not habituated to drink clarified juices.

Fresh pulp was associated with terms such as not very tasty, thick and consistent, cooked flavor, and dreggy, showing an average score of 6.0, which did not significantly differ from that of juices concentrated by reverse osmosis or clarified juices. With respect to appearance, the juice concentrated by osmotic evaporation presented the highest mean value (7.4) and the other samples did not differ from each other in this characteristic.

Despite standardization of the soluble solid content by adding sugar, the fresh pulp was characterized as acidic or slightly sweet. According to Rousmans et al. (2000), the greater acceptance of the sweet taste is explained by the evolution of species, which resulted in physiological systems that give the feeling of pleasure in response to certain gustatory stimuli [46]. For example, molecules that have energy nutrients such as sucrose generate pleasant feelings. On the other hand, responses to stimuli related to the presence of toxins are generally associated with bitter taste.

De Marchi et al. (2009) studied the acceptability of a natural passion fruit beverage using different levels of passion fruit pulp and sucrose and observed that juices formulated with higher sucrose concentrations (between 10 and 17.5%) showed the highest acceptability scores [47].

Consumers’ descriptions of the samples were highly related to the differences in juice characteristics (χ2 = 184.97, *p* < 0.0001). Correspondence analysis was applied to obtain a synthetic map of the relationship between samples and consumers’ descriptions. The first two dimensions accounted for 92.04% variability in the experimental data (Figure 5).

## 4. Conclusions

Membrane technology not only helps preserve the nutritional integrity of blackberry juice but also enables the creation of value-added, high-quality products that meet the diverse preferences of today’s health-conscious consumers.

Osmotic evaporation is a promising technology for concentrating fruit juices due to its ability to preserve flavor and nutrients. However, like low hydrophobicity membrane materials, it does have some constraints, that limit its widespread use and efficiency. Advances in membrane technology, materials, and process optimization are key to overcoming these challenges and making membrane-based concentration more viable for large-scale, cost-effective juice production.

The coupling of reverse osmosis and osmotic evaporation was shown to be a potential alternative to the concentration of clarified blackberry juice, resulting in a final product with 55.5 °Brix of soluble solid content.

## Figures and Tables

**Table 1:** Physicochemical characteristics of blackberry juice before and after the microfiltration process.

Sample	pH	Titratable Acidity (g∙kg^−1^) *	Total Soluble Solids (°Brix)	Total Solids (g∙kg^−1^)	Total Anthocyanins (mg∙kg^−1^) **	Antioxidant Activity (μmol Trolox∙g^−1^)
Fresh pulp	3.00 ± 0.00	12.40 ± 0.50 ^a^	7.00 ± 0.00 ^b^	84.50 ± 0.40 ^b^	47.68 ± 2.15 ^b^	9.08 ± 0.12 ^b^
Feed	2.99 ± 0.00	12.00 ± 0.10 ^a^	7.00 ± 0.10 ^b^	84.90 ± 0.30 ^b^	49.05 ± 1.25 ^b^	9.43 ± 0.05 ^b^
Retentate	3.00 ± 0.00	11.70 ± 0.10 ^a^	8.00 ± 0.10 ^a^	99.30 ± 0.90 ^a^	67.58 ± 1.80 ^a^	12.31 ± 1.40 ^a^
Clarified juice	3.00 ± 0.00	11.30 ± 0.40 ^a^	6.50 ± 0.00 ^c^	77.30 ± 0.50 ^c^	31.21 ± 0.05 ^c^	5.54 ± 0.10 ^c^

Different letters in the same column indicate significant difference between different samples (*p* ≤ 0.05), determined by Tukey’s test. * Expressed in g/100 g citric acid. ** Expressed in cyanidin-3-glucoside.

**Table 2:** Physicochemical characteristics of concentrated blackberry juice obtained by coupling reverse osmosis and osmotic evaporation.

Analysis	Feed (Clarified Juice)	Reverse Osmosis	Osmotic Evaporation
pH	3.00 ± 0.00 ^a^	2.97 ± 0.00 ^a^	2.96 ± 0.00 ^a^
Total soluble solids (°Brix)	6.50 ± 0.00 ^c^	24.60 ± 0.00 ^b^	55.50 ± 0.00 ^a^
Total solids (g∙kg^−1^)	77.30 ± 0.50 ^c^	277.60 ± 0.80 ^b^	569.40 ± 1.10 ^a^
Titratable acidity (g∙kg^−1^) *	11.30 ± 0.00 ^c^	38.40 ± 0.40 ^b^	77.50 ± 0.00 ^a^
Total anthocyanins (mg∙kg^−1^) **	31.21 ± 0.05 ^c^	130.32 ± 0.53 ^b^	192.87 ± 0.52 ^a^
Antioxidant activity (µmol TE∙g^−1^)	5.54 ± 0.10 ^c^	22.32 ± 0.72 ^b^	42.85 ± 0.19 ^a^

Different letters in the same line indicate significant difference between different samples (*p* ≤ 0.05), determined by Tukey’s test. * Expressed in g/100 g citric acid. ** Expressed in cyaniding-3-glucosides.

**Table 3:** Overall liking and appearance scores of blackberry juices.

Samples	Overall Liking	Appearance
Fresh pulp	6.0 ^bc^	6.8 ^b^
Clarified juice	5.6 ^c^	6.7 ^b^
Juice concentrated by reverse osmosis	6.2 ^b^	6.7 ^b^
Juice concentrated by osmotic evaporation	6.9 ^a^	7.4 ^a^

Different letters in the same column indicate significant difference between different samples (*p* ≤ 0.05), determined by Fisher’s test.

## Data Availability

Data supporting the findings of this study are available within the article.
